# Allele-specific expression assays using Solexa

**DOI:** 10.1186/1471-2164-10-422

**Published:** 2009-09-09

**Authors:** Bradley J Main, Ryan D Bickel, Lauren M McIntyre, Rita M Graze, Peter P Calabrese, Sergey V Nuzhdin

**Affiliations:** 1 Section of Molecular and Computational Biology, Department of Biological Sciences, University of Southern California, Los Angeles, California 90089, USA; 2 Departments of Molecular Genetics and Microbiology and Statistics, University of Florida, Gainesville, Florida 32610-1399, USA

## Abstract

**Background:**

Allele-specific expression (ASE) assays can be used to identify *cis*, *trans*, and *cis*-by-*trans *regulatory variation. Understanding the source of expression variation has important implications for disease susceptibility, phenotypic diversity, and adaptation. While ASE is commonly measured via relative fluorescence at a SNP, next generation sequencing provides an opportunity to measure ASE in an accurate and high-throughput manner using read counts.

**Results:**

We introduce a Solexa-based method to perform large numbers of ASE assays using only a single lane of a Solexa flowcell. In brief, transcripts of interest, which contain a known SNP, are PCR enriched and barcoded to enable multiplexing. Then high-throughput sequencing is used to estimate allele-specific expression using sequencing counts. To validate this method, we measured the allelic bias in a dilution series and found high correlations between measured and expected values (r>0.9, p < 0.001). We applied this method to a set of 5 genes in a *Drosophila simulans *parental mix, F1 and introgression and found that for these genes the majority of expression divergence can be explained by *cis*-regulatory variation.

**Conclusion:**

We present a new method with the capacity to measure ASE for large numbers of assays using as little as one lane of a Solexa flowcell. This will be a valuable technique for molecular and population genetic studies, as well as for verification of genome-wide data sets.

## Background

Genotype-phenotype mapping is a fundamental aim of biological science. This is critical for many goals such as understanding of how genetic architecture shapes phenotypic variation and adaptation [1-3], and more specific aims such as deciphering how genetic variation in humans may affect response to treatment [4,5]. Many genetic variants resulting in phenotypic differences are mediated through changes in gene expression. Thus, analyzing gene expression allows us to better understand genotypic variation. Variation in gene expression can be due to polymorphisms both at the gene locus (*cis*) and in other genes that influence its expression (*trans*), as well as the non-additive interactions between the two (*cis*-by*-trans*) [6]. Furthermore, epigenetic mechanisms [7], chromatin conformation [8], copy number variation [9,10] and microRNA [11] all play important roles in the transcription of a given gene. By partitioning regulatory variation into *cis, trans*, and *cis*-by-*trans*, we can identify their respective contributions to changes in gene expression and potentially how expression levels evolve within the genomes of complex organisms [12,13].

Allele-specific expression (ASE) studies have introduced a creative method to uncover the respective contributions of *cis*- and *trans*-regulatory variation [12,14-17]. First, total expression differences are measured from a pooled sample of two homozygous lines. Then, *cis*-regulatory variation is estimated from the allelic imbalance (unequal expression of alleles) in the corresponding F1 heterozygote, where each allele is regulated by the same *trans*-factors [18]. *Trans *can then be inferred from the total expression differences that are not explained by *cis*. Of course, these inferences about *cis*- and *trans*-regulatory variation can be complicated if *cis*-elements and *trans*-factors interact non-additively [17,19,20]. Allelic imbalance in non-imprinted genes has been shown to be common in mice, maize and humans [18,21,22]. Also, a few studies have investigated *cis*- and *trans*-regulatory variation within and between species of *Drosophila*. Measuring ASE, Wittkopp *et al*. reported that *cis*-regulatory variation plays a predominant role in divergent gene expression between *D. melanogaster *and *D. simulans *[15].

**Figure 1:**
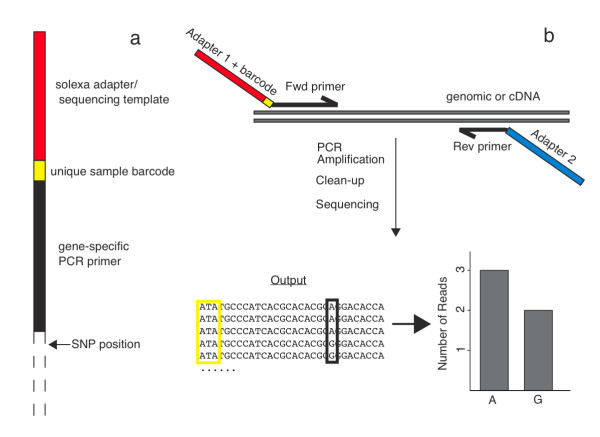
**ASE using Solexa**. Summary of sample preparation: (a) The forward primer is designed to anneal 1 bp upstream of the known SNP. The 5' tail contains a unique barcode and adapter sequences necessary for hybridization to the Solexa sequencing platform. (b) Model of target enrichment and allele-specific expression represented as sequencing coverage per allele.

**Figure 2:**
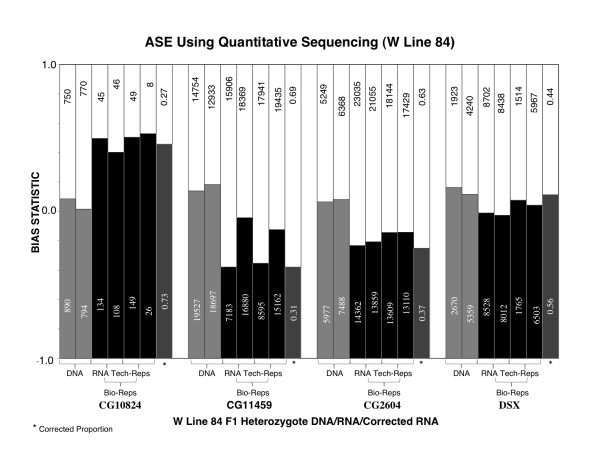
**Count-based ASE assays**. Allele-specific expression represented as sequencing reads per W-allele (black) and tester allele (white). The sequencing coverage per allele is shown on each bar and the corrected RNA (*) is represented as a proportion of each allele. The y-axis is the proportion of the difference (allele1- allele2)/(allele1 + allele2).

**Figure 3:**
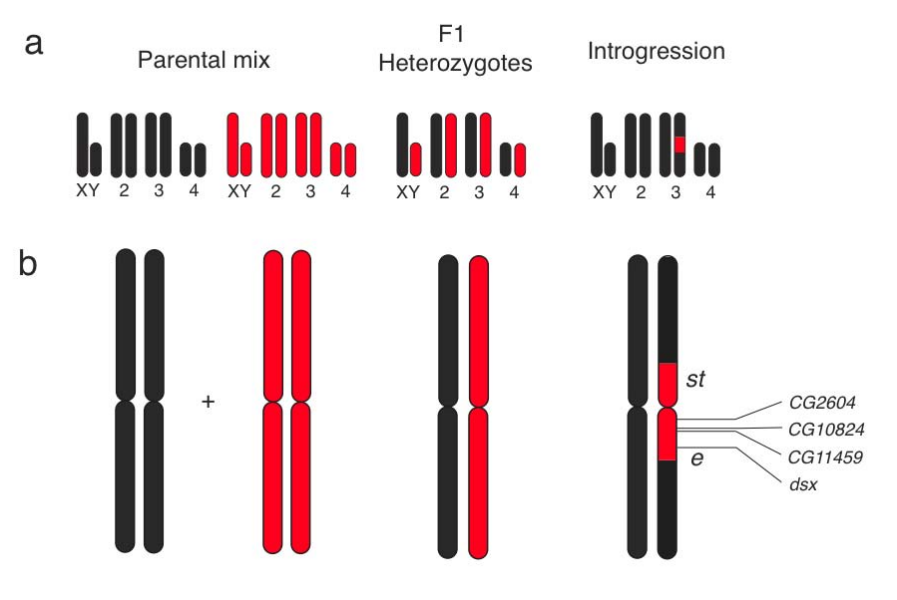
**Nucleic acid samples**. The three nucleic acid sample types used in this study: the parental mix, F1 heterozygote, and introgression lines. (a) Representation of all chromosomes for each genetic background assayed. Red represents the W line and black represents the tester line. (b) Zoom-in of chromosome 3 for each genotype. All genes analyzed are between the genetic markers *scarlet *(*st*) and *ebony *(*e*).

Allele-specific expression has been measured using various targeted approaches including reverse transcription-PCR (asRT-PCR; [23]), and pyrosequencing [15,24]. Genome-wide approaches have also been used including serial analysis of gene expression (SAGE) [25,26], massively parallel signature sequence (MPSS)[27,21], and microarray-based methods [22,28]. Here, we introduce a simple approach to ASE assays that combines a targeted approach to gene expression assays with the power of high-throughput sequencing. In brief, transcripts of interest (containing a known SNP) are PCR enriched and barcoded to enable large-scale multiplexing. Using this approach, we sequence only regions of interest and allele-specific read counts are used to estimate ASE for large numbers of samples using a single lane of a Solexa flowcell (Figure 1 and 2). To demonstrate its application, we investigated variation in gene expression in a set of five *Drosophila simulans *genes. Using a common tester line, we measured ASE in an equal mix of homozygotes (parental mix), a heterozygote, and an introgression (Figure 3). By analyzing changes in ASE under these different regulatory conditions, we elucidate the respective contributions of *cis, trans*, and *cis*-by-*trans *interactions on variation in gene expression. Furthermore, we tested for within-species variation in *cis *and *trans *by comparing trends in ASE between six highly inbred lines collected from a single population.

## Results and discussion

### Digital ASE assay

In this study, we introduce an accurate approach to allele-specific expression assays that relies on read counts generated from Solexa sequencing. For each gene of interest, a single nucleotide polymorphism (SNP) within the mRNA transcript was identified that differs between the lines of interest and our tester line. We then designed a PCR primer that annealed immediately flanking the 5' end of the SNP and another primer that annealed 200-300 base pairs downstream (Figure 1). In the primer design, we included adapter sequences, provided by Illumina, as 5' tails to allow these PCR products to be sequenced on the Solexa platform without additional steps. When we planned on analyzing more than one sample per gene, a unique forward primer was ordered for each sample that contained the common elements described above, plus a unique one to three base pair barcode sequence in the 5' tail that will allow for individual sample identification (Figure 1a). We used a touchdown PCR cycling program to enrich each sample for our target region and then purified the amplicons using gel extraction. We then pooled the purified samples in large numbers (in our case, 300) and sequenced them in parallel on a single lane of a Solexa flow cell. The resulting reads were analyzed by assigning each read to a specific gene based on homology and sample based on the unique barcode (see methods). We can then compare the number of reads containing each SNP to have a digital representation of ASE (Figure 1b and 2).

In all comparisons, both alleles were amplified in the same reaction and thus utilized the same reagents. As a result, each allele should theoretically maintain the same relative abundance throughout amplification. However, this may not be the case if small differences in primer hybridization or polymerase efficiency exist between alleles. We can control for this error in amplification by analyzing heterozygous DNA samples, where the actual allele frequency is 50:50, and then correcting each RNA sample by the difference observed in the heterozygote ([15] and Additional file 1). DNA samples from each mixed sample were also analyzed in order to correct for both allele-specific amplification error and differences in cell number between the individuals extracted from each parental line [15]. We report allelic imbalance as: the proportion of reads that are differentially expressed ((allele1 - allele2)/(allele1 + allele2)). This approach allows us to easily estimate the binomial sampling error. If there is no difference between alleles, the bias is zero, while the bias is positive if the first allele is favored and negative if the second allele is favored.

**Figure 4:**
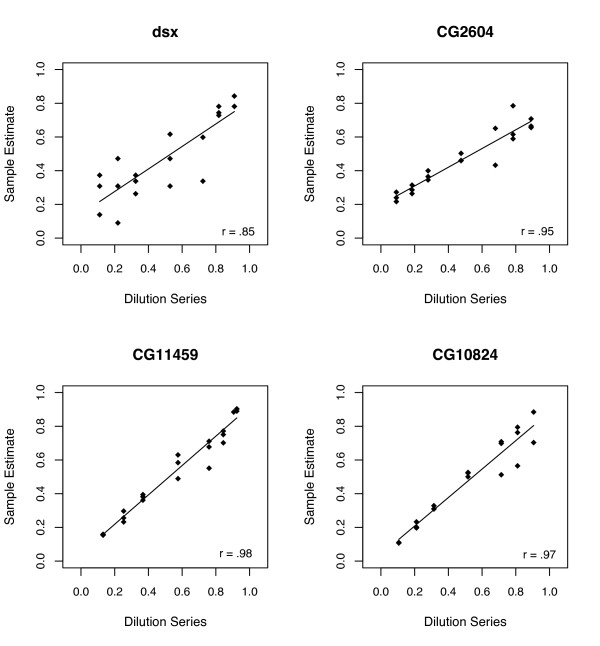
**Verification of ASE assays using Solexa**. Each data point represents one of three replicate dilutions analyzed at each step of the dilution series (9:1, 8:2, 7:3, 5:5, 3:7, 2:8, 1:9). The distribution of sequencing reads within each gene is demonstrated as follows: mean ± SE (n = 21). DSX = 11.3 ± 3.8, CG2604 = 2,478.3 ± 288.8, CG10824 = 2,756.4 ± 333.5, CG11459 = 11,578.6 ± 2515.1.

To verify the accuracy of Solexa and the normalization procedure, we created three replicate dilution series using genomic DNA from the tester line and an experimental line (W line 84). Then we estimated the allelic bias at each step of the dilution (in multiplex) using a separate sequencing lane. All four genes demonstrated a strong correlation (r >0.9 p < 0.001) between the expected and observed allelic bias (Figure 4). The gene *dsx *had a relatively low correlation, because there was very limited sequencing coverage for all samples within this gene. Thus, the Solexa sequencing output can be used to accurately measure the relative abundance of alleles in a given sample.

By analyzing technical variation and correlation coefficients in the verification experiment, it appears that coverage on the order of a few hundred reads is sufficient to yield reproducible results (Figure 4 and Additional file 1). Thus, increased biological replication should be favored over sequencing coverage beyond a few hundred reads (Additional file 1: Table S3). In this study, coverage varied widely between genes (Additional file 1: Table S1), while coverage of individual assays within each gene was similar (Additional file 1: Table S3). This is most likely due to variation in initial transcript abundance, variation in amplification efficiency between genes and the resulting uneven pooling of DNA between genes. Therefore, we suggest that if the pooling of DNA is done carefully based on the concentration of each purified sample or PCR amplicon band intensity, the sequencing coverage can be more evenly distributed. This will also increase the number of high-quality ASE assays that can be performed on a single lane of Solexa.

The cost per base of sequence using Solexa is very low, so for large-scale projects, the preparation costs such as barcoded primers become the limiting economic factor. To address these concerns, we suggest one of the following approaches depending on the type of project: For studies where many assays are performed with a few select genes, barcode costs can be significantly reduced if paired-end sequencing reads are combined with multiplicative barcoding. For example, instead of ordering 900 barcoded adapters for a given gene, we can create 900 unique barcode combinations using only 30 barcoded forward primers and 30 barcoded reverse primers. For studies involving large gene sets, barcoded adapter sequences can be added to typical gene-specific annealing primers before PCR using single-strand ligation. Using this approach, barcoded adapter sequences can be used in multiple experiments. We have shown that Solexa can effectively estimate ASE using this targeted approach and we mention these additional modifications to allow easier adaptation for future researchers.

### Error analysis

To understand the error in quantifying ASE, we looked at sources of variation at multiple levels. First, we estimated sequencing error at the SNP position by identifying the proportion of reads with an incorrect base-call. Sequencing error was well below 2% for most of the genes. Furthermore, this error rate did not seem to change when the position of the SNP within the read shifted due to barcode lengths changing from one to three base pairs (Additional file 1: Table S2). Second, we estimated the binomial sampling error and found that this error quickly becomes negligible with the high coverage obtained with this method (Additional file 1: Table S3). Third, we assessed the error introduced by the method, such as polymerase and reverse transcription error by comparing allelic imbalance between technical replicates (cDNA templates created separately from the same RNA pool). This technical variation was considerably more than binomial sampling once coverage was over a few hundred reads (Additional file 1: Table S3). And finally, to analyze overall variation in expression estimates, including dynamic gene expression *in vivo*, we compared ASE estimates between biological replicates (separately collected material from the same genetic cross). The biological variation was greater than technical variation in this study, but an accurate assessment of the relative magnitude of technical and biological variance is beyond the scope of this study and thus, both should be considered when designing future experiments (Additional file 1: Table S3).

### *Cis*- and *trans*-regulatory variation within species

**Figure 5:**
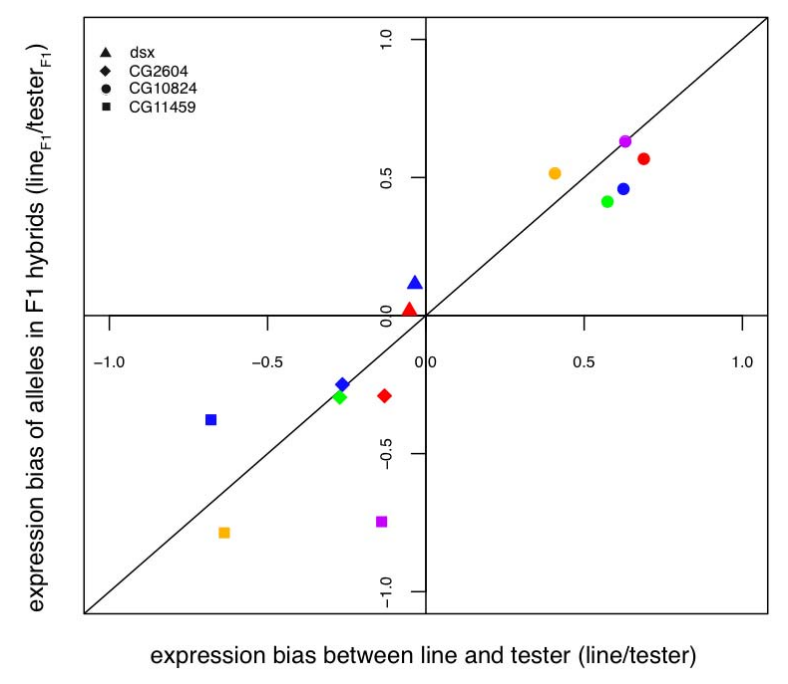
**Intraspecific regulatory variation due to *cis *and *trans***. Expression bias is the proportion of the difference between alleles: (allele1- allele2)/(allele1 + allele2). The parental mix is plotted on the x-axis and the F1 heterozygote is plotted on the y-axis. A 1:1 relationship would indicate 100% *cis *and a slope of zero would indicate 100% *trans*. A 1:1 line is shown for reference. The vast majority of the expression differences between the W lines and the tester line are attributed to *cis*, for the four genes and six lines tested (regression coefficient = 0.91 ± 0.13). Color code: line 84 - blue, line 181 - red, line 147 - purple, line 192 - orange, and line 68 - green.

We used six highly inbred lines of *D. simulans *from Winters, CA (W Lines) and a *scarlet ebony *(*st e*) tester line in this study. For each W line, we compared expression to the tester line in a parental mix (*i.e*. an equal mix of homozygous tester and W line flies), the related F1 heterozygote, and a corresponding introgression (see methods for details) (Figure 3). This experimental design allows us to assess intraspecific regulatory variation in *cis*, *trans *and *cis-by-trans*. To do this, we estimated the overall expression differences in four genes in the aforementioned parental mix. Then, we determined *cis*-regulatory variation from the allelic imbalance present in the related F1 heterozygote, where *trans*-factors affect each allele equally. We can then infer *trans *from the overall expression difference that is not explained by *cis*. In the four genes analyzed, *cis*-regulatory variation explains the majority of the overall divergence in gene expression between the tester line and the W lines (Figure 5, regression coefficient = 0.91 ± 0.13, [12]). It should be noted that this is a small gene set and most of these genes have been shown to be variable within-species (see methods). Thus, we do not consider this result to be a reflection of the genome as a whole. One explanation for the small contribution of *trans *in these genes is that *trans*-variation has an increased potential for pleiotropic effects, some of which may be deleterious and removed by purifying selection in the W lines tested.

Gene expression is a result of *cis*-regulatory elements interacting with *trans*-regulatory proteins. If there is variation in both *cis*-elements and *trans*-proteins, there is the potential for non-additive interactions (*cis*-by-*trans*) [29]. To test for *cis*-by-*trans*, we compared allelic imbalance in the heterozygote and the introgression within each W line (Figure 3). If all genes act additively, allelic imbalance in the heterozygote and introgression should be equal because the *cis*-regulatory elements for each allele and the trans-factors within each genotype are identical. If allelic imbalance between these genotypes is not equal, that difference is due to *cis*-by-*trans*. We lacked the statistical power to individually detect these *cis*-by-*trans *interactions, but we were able to test for a systematic shift in expression between these genotypes across all genes and W lines. We hypothesized that if the *cis*-elements and *trans*-factors within the tester line and the W lines co-evolved, non-additive interactions would be relatively common and therefore we might detect significant *cis*-by-*trans *effects across all genes and lines. To test for this, we analyzed the relationship between heterozygous and introgression ASE values for the 6 W lines and 4 genes using a linear model. The regression coefficient is not significantly different from one (p = 0.5) and thus, there is no systematic *cis*-by-*trans *interactions detected within our sample population and selected genes. This result, together with recent findings seems to be indicating that at least within *Drosophila*, epistasis may be rare or is small in magnitude [17]. This may also be due to the lack of population structure in *Drosophila*, which would hinder the co-evolution of divergent *cis*-elements and *trans*-factors.

To compliment our previous estimates of *cis *and *trans *between the tester line and the W lines, we measured variation in *cis *between W lines in order to give additional insight into the type and magnitude of regulatory variation that may be segregating in natural populations. To identify this variation we tested for a significant effect of a given W line on the overall estimate of *cis *and *trans*. Using this approach we were unable to detect significant variation between lines (p = 0.31). We did however find evidence for variation between the W lines and the tester line suggesting that genetic variation may be more common between populations (Figure 2).

## Conclusion

We have presented a new method that uses Solexa to accurately measure ASE for hundreds of samples using as little as one lane of a Solexa flowcell. This will be a valuable technique for analyzing a few genes for many individuals or under many conditions, measuring a large selection of genes for a few individuals, and verifying ASE estimates from genome-wide expression assays.

## Methods

### Fly strains and genotypes

*D. simulans *lines used in this study were collected from the Wolfskill orchard in Winters, CA and inbred for a minimum of 20 generations to create highly inbred lines (W lines) [30]. A *D. simulans *tester line containing the visible mutations *scarlet (st) *and *ebony (e) *(*st *((chr3L: 15833418-15836165), *e *(chr3R: 4397293-4404648)) was obtained from the Drosophila stock center at UCSD (stock number 14021-0251.041) and inbred for over 20 generations. We analyzed ASE in a parental mix, heterozygote, and introgression for each W line against the common tester line (lines: 84, 181, 147, 201, 68, 192) (Figure 3). To create the parental mix, homozygous male flies from a given W line were homogenized together with homozygous male flies from the tester line. The heterozygous genotype was the F1 male progeny from a cross between a given W line male and two virgin tester line females. And finally, to construct introgression lines, F1 heterozygous females were created from an initial cross between a given W line female and *st e *male. Then they were backcrossed to the *st e *line. After each generation, wild-type females were selected (heterozygous genotype at both markers) for subsequent backcrossing. This process was repeated for a minimum of 20 generations to create an introgression line. Thus the introgression line is heterozygous for approximately 12 Mb between the genetic markers *st *and *e *on the third chromosome, while the remainder of the genome is homozygous for the *st e *line (Figure 3). Two replicate introgression lines were created in parallel for each W line (introgression A and B) to control for expression differences due to different introgression cutoff points flanking the visible markers. Reciprocal crosses were not analyzed, but the effects of genomic imprinting, the only type of parent-of-origin effects that would impact ASE, are likely rare or do not exist in adult Drosophila [31]. All flies were reared at ~25°C with a 12 h:12 h light cycle.

### Gene selection

We chose a set of genes within the introgressed region on chromosome three for further analysis (Figure 3b). Genes were chosen based on current interest in the lab (*dsx*) and previous analyses showing them to vary within species (*Cyp21c, CG10824, CG2604*) [30] or between species (*CG11459*) [15]. We resequenced most of the coding region of each gene and then designed our gene-specific primers based on the location of a single nucleotide polymorphism (SNP) between the W lines and the *st e *tester line. The *Cyp21c *primers non-specifically amplified a homologous gene (*Cyp4ac3*), thus it was not analyzed further.

### Extraction of nucleic acids and cDNA synthesis

We extracted DNA and RNA in parallel from 14 whole-body adult male flies for each sample using a modified protocol for Promega's SV Total RNA Extraction Kit [15]. Experimental samples included a parental mix and a heterozygous DNA sample, along with cDNA from the parental mix, F1, and introgression A and B (see above) for each W line. We included replicate biological samples (separately collected material from the same genetic cross) for all experimental samples involving W line 181 and 84. Also, we included technical replicate (cDNA templates created separately from the same RNA pool) samples for all biological samples involving W lines 84 (Additional file 3). To extract nucleic acid from the parental mix, we homogenized seven homozygous male flies from a given W line together with seven homozygous male flies from the *st e *line. The heterozygous and the introgression genotypes were extracted from 14 male progeny. Before extraction, adult male flies (three to five days old) were snap-frozen in liquid nitrogen in the morning and stored at -70°C until extraction. Briefly, we passed the supernatant from fly homogenate through an affinity column optimized for binding DNA. Then the flow-through was run through another column optimized to bind all RNA. We then treated RNA columns with DNAse, followed by subsequent washing steps and elution. Using Applied Biosystem's (AB) High-Capacity cDNA Reverse Transcription Kit we immediately synthesized cDNA from the RNA samples using AB's standard protocols with RNAse inhibitor. Following extraction, we stored all cDNA and DNA samples at -70°C until further preparation.

### PCR and purification

PCR primers for the ASE assay were designed such that one annealed immediately flanking the five prime (5') end of the SNP to be sequenced and the other, 200-300 base pairs downstream (3'). Then we modified each primer design with a custom 5' tail consisting of Solexa adapter sequences. Furthermore, to allow for multiplexing, a sample-specific barcode (one to three base pairs) was added to the design between the adapter and the annealing primer (Figure 1 and Additional file 2).

All PCR reactions were performed using Finnzymes Phusion High-Fidelity DNA Polymerase and a touchdown cycling program stepping down 1° each cycle from 70°C to 60°C, followed by an additional 25 cycles at 60°C annealing. Samples were run on a 2% agarose gel for amplicon size confirmation and agarose gel purification. PCR products were gel purified using Qiagen's Qiaex II Gel extraction kit and standard protocols. Following purification, a portion of each of the 300 PCR enriched samples was pooled together, ethanol precipitated and then resuspended to a concentration of 10 nM. The pooled sample was sequenced at Cornell's core sequencing center using a custom primer.

### Data analysis

Using a custom Perl script, the 6 million reads generated from a single lane of a Solexa flow cell were assigned to one of our five genes based on the first eight base pairs (allowing for one mismatch) of the annealing primer. Within each gene pool, the reads were then separated into 60 unique ASE assays using the one to three base pair sample-specific barcodes (Figure 1). We then checked for a known sequence surrounding the SNP, including five base pairs 5' of the SNP and eight base pairs 3' of the SNP. If these 13 base pairs (5 bp pre-SNP + 8 bp post-SNP) could not be identified (allowing for a single mismatch) the reads were discarded. We then scored each read for the nucleotide in the SNP position to determine the allele-specific read count (Additional file 3).

To determine the respective contributions of *cis*- and *trans*-regulatory variation, we compared ASE in the parental mix flies and the heterozygous flies. ASE differences in the parental mix reflect total variation in gene expression, including *cis *and *trans*. In contrast, *trans *is shared between alleles in the heterozygote, thus only *cis *contributes to ASE differences in this genotype. As a result, *trans *can be inferred from the percent of the total variation estimated in the parental mix (*cis+trans*) that is not explained by the variation estimated in the heterozygote (*cis*). To perform this test this we used the model: Y_ij _= μ+BX_ij_+e_ij _where for the i^th ^W line and the j^th ^gene, Y is the estimated difference between alleles in the heterozygote (*cis*) and X is the total difference in expression estimated from the parental mix. Then, we estimated *trans*-regulatory variation from the percent of the overall expression differences found in the parental mix that are not explained by this model (Figure 5). Missing data points are due to lack of a SNP within a given gene and W line.

To identify *cis*-by*-trans *interactions (*i.e*. non-additive effects), we tested whether the allelic bias in heterozygotes was significantly different from the allelic bias in introgressions. To examine this effect, we fit the model: Y_ij _= μ+BZ_ij_+e_ij _where for the i^th ^W line and for the j^th ^gene, Y is the estimated difference between alleles for the heterozygote and Z is the estimated difference between alleles for the introgression line. The *cis*-regulatory elements for each allele in the heterozygote are identical to the *cis*-regulatory elements in the introgression, hence the allelic bias cannot vary due to *cis *between these genotypes. Furthermore, although the *trans*-factors change between genotypes (heterozygous W/*st e *to homozygous *st e*) the *trans*-factors are shared equally between alleles within each genotype and thus, differences in *trans *cannot contribute to allelic bias. As a result, any change in allelic bias between these genotypes must be due to *cis*-by-*trans *interactions.

To test for natural variation (*i.e*. variation between W lines) in the relative contribution of *cis *and *trans*, we fit the model Y_ij _= μ+B_1_X_ij_+B_2_L_i_+e_ij _where for the i^th ^line and the j^th ^gene, Y is the average bias between alleles for the parental mix, and X is the average bias between alleles for the heterozygote. We then tested for the effect of line.

To verify the accuracy of ASE assays using Solexa, we used a separate lane of a flow cell to analyze three replicate dilution series (1:9, 2:8, 3:7, 5:5, 7:3, 8:2, 9:1). These dilutions were created using genomic DNA extracted separately from homozygous W line 84 and the homozygous *st e *tester line (Figure 4). Each of the four genes analyzed in this study were tested for the ability to accurately detect known allele frequencies. Heterozygous DNA from a cross between these lines was also analyzed in parallel to correct for allele-specific amplification bias ([15] and Additional file 1). To correct for differences in starting concentration of the homozygous DNA used for the dilution series, we corrected the expected values by the average allelic bias found in the 1:1 mix [15]. Correlations between the known dilution and the measured ASE using Solexa were estimated separately for each gene using Pearson's correlation coefficient [32].

## Authors' contributions

BJM conceived the method and performed the experiments. SVN BJM RMG designed the experiment. RDB SVN BJM LMM PPC analyzed the data. BJM RDB LMM SVN wrote the paper. All authors read and approved the final manuscript.

## Supplementary Material

Additional file 1 **Supplemental materials**. "Supplemental material" Click here for file

Additional file 3 **Sequencing counts**. "Raw counts" All sequencing counts for each sample. Click here for file

Additional file 2 **Detailed protocol**. "ase using solexa protocol " Detailed Protocol Click here for file
