# Supplementary material for: Allele-specific expression assays using Solexa
Source: BMC Genomics. 2009 Sep 9;10:422. doi: 10.1186/1471-2164-10-422 (PMC2749874; doi:10.1186/1471-2164-10-422)
Supplement: Additional file 1 — Supplemental materials. "Supplemental material" [file 1471-2164-10-422-S1.doc]

**Allele-specific expression assays using Solexa - Supplemental material**

**Table 1.** Solexa Single Lane Sequencing Coverage Per Gene

Coverage with relaxed alignment criteria around the SNP

(5bp Pre-SNP and **3**bp's post-SNP)

| **Line** | **Reads** | **%** |
| --- | --- | --- |
| CG11459 | 2,088,931 | 35% |
| CG2604 | 1,434,025 | 24% |
| DSX | 963,692 | 16% |
| CG10824 | 107,315 | 2% |
| *CG4120 | 1,065,503 | 18% |
| unable to align | 340,501 | 5% |
| Total | 5,999,967 | 100% |

Coverage with increased alignment criteria around the SNP

(5bp Pre-SNP and **8**bp's post-SNP)

| **Line** | **Reads** | **%** |
| --- | --- | --- |
| CG11459 | 2043861 | 34% |
| CG2604 | 1401941 | 23% |
| DSX | 924548 | 16% |
| CG10824 | 103581 | 2% |
| *CG4120 | 9109 | 0.20% |
| unable to align | 1516927 | 25% |
| Total Reads | 5999967 | 100% |

*CG4120 (Cyp21c) was thrown out of analysis because of non-specific amplification on the 2nd (Cyp4ac) and on the 3rd Chromosome (Cyp21c genes).

**Table 2**. Sequencing Error and Position Effect

Values are the proportion of non-SNP base pairs at different positions in the sequencing read as the barcode length varied.

| Position in Read | *CG10824 (A/G SNP) | CG2604 (C/T SNP) | DSX * (A/G SNP) | CG11459 (C/T SNP) |
| --- | --- | --- | --- | --- |
| SNP pos. #20 | 0.0079 | 0.002 | 0.0228 | N/A |
| SNP pos. #21 | 0.0089 | 0.002 | 0.0236 | N/A |
| SNP pos. #22 | 0.0052 | 0.002 | 0.0117 | 0.0008 |
| SNP pos. #23 | N/A | N/A | N/A | 0.0005 |
| SNP pos. #24 | N/A | N/A | N/A | 0.0008 |

* A/G SNP's both have a consistent bias for miscalling C's over T's (data not shown).

**Table 3.** Estimation of the mean binomial sampling variance, mean experimental variance, and mean biological variance for each gene and type of assay.

| Gene | type | Mean number of reads | Mean Binomial sampling | Mean Technical | Mean Biological |
| --- | --- | --- | --- | --- | --- |
|  |  |  | s ^2^ | s^2^ | s^2^ |
| *CG10824* | parental mix | 2,173.50 | 0.00064 | No data | 0.0117 |
| *CG10824* | Heterozygote | 165.2 | 0.00755 | 0.0034 | 0.0023 |
| *CG10824* | introgression | 903.8 | 0.00242 | 0.0044 | 0.0045 |
| *CG11459* | parental mix | 28,964.30 | 0.00003 | No data | 0.0033 |
| *CG11459* | Heterozygote | 29,867.80 | 0.00004 | 0.0308 | 0.0155 |
| *CG11459* | introgression | 20,913.10 | 0.00011 | 0.0021 | 0.0293 |
| *CG2604* | parental mix | 23,445.80 | 0.00011 | No data | 0.0008 |
| *CG2604* | Heterozygote | 26,801.50 | 0.0001 | 0.0002 | 0.0033 |
| *CG2604* | introgression | 22,834.10 | 0.00008 | 0.0002 | 0.0058 |
| *dsx* | parental mix | 11,903.50 | 0.00027 | No data | 0.0284 |
| *dsx* | Heterozygote | 13,466.30 | 0.00018 | 0.0002 | 0.0016 |
| *dsx* | introgression | 17,058.70 | 0.00017 | 0.0042 | 0.0032 |
|  |  |  |  | **0.0057** | **0.0091** |
| estimate of the variance = s^2^ | |  | | | |

The biological and technical variance values were obtained from the mean of the appropriate pairwise variances from the replicates of lines 181 and 84. Binomial sampling variance was estimated from the mean number of reads from the same samples.

Correction of RNA using ASE in Het DNA

| DNA |  |
| --- | --- |
| Allele1 | d1 |
| Allele 2 | d2 |
| RNA |  |
| Allele 1 | r1 = d1 × e1 |
| Allele 2 | r2 = d2 × e2 |
| Unobserved expression |  |
| Allele 1 | e1 |
| Allele 2 | e2 |
|  |  |

Due perhaps to bias in the amplification of each allele during PCR or a difference in size of the flies from each genotype in the “parental mix”, the ratios d1/(d1+d2) may not approximate 0.5. We assume that this bias, whatever its reason, is the same for DNA and RNA. Therefore, the observed RNA counts are a product of the DNA counts, which incorporate this bias, and some unobserved expression value, which reflects the actual amount of each allele.  Thus, we can infer the actual RNA counts (unobserved expression) based on the observed RNA (r1, r2) and DNA (d1,d2) counts (see chart above).
