# Supplementary material for: Allele-specific expression assays using Solexa
Source: BMC Genomics. 2009 Sep 9;10:422. doi: 10.1186/1471-2164-10-422 (PMC2749874; doi:10.1186/1471-2164-10-422)

**Allele-specific expression using Solexa protocol**

1) Identify a SNP between two parental lines.

2) Design gene specific 18-20bp annealing primers as follows: forward primer flanking the 5' end of the SNP such that the base immediately following the 3’ end of the primer is the SNP, the second 200-300bp's downstream from the SNP.

3) Check primer design and verify that no additional SNP's occur within the gene specific annealing primers.

4) Create barcodes for each unique sample (we used 1-3 bp's).

5) Obtain Solexa adapter sequences A1 and A2 available upon request from Illumina.

6) Identify Solexa Adapter 1 sequence (A-1) (note: be sure to include the additional adapter sequence added during PCR enrichment step. The A1 adapter alone will not be sufficient). Solexa Adaptor 2 sequence should be as given. (Figure 1p)

Design forward primers with modified 5' tails, starting 5' to 3' as follows: Solexa Adapter-1 sequence, barcode, forward primer from step 2.

7) Design reverse primers with modified 5' tails, starting 5' to 3' as follows: Solexa Adapter-2 sequence, reverse primer from step 2.

8) Design a custom Solexa sequencing primer to anneal immediately flanking the barcode sequence on the primer from step 7 (Figure 1p).

9) Extraction of Nucleic Acids:

- DNA and RNA were isolated from 14 individual flies using a protocol modified by Wittkopp (2004) for Promega's SV Total RNA Extraction Kit. In brief, RNA was isolated as described with DNAse treatment of column-bound RNA prior to elution. The DNA, normally discarded with the DNA affinity column, was washed and eluted from the column using water. Nucleic acid quantification was carried out using a Nanodrop. All samples were stored at -70 C.

10) cDNA Synthesis:

-cDNA was synthesized from total RNA within 24 hours of isolation using the High-Capacity cDNA Reverse Transcription Kit with RNAse Inhibitor (Applied Biosystems) according to the manufacturer's protocol. All cDNA samples were stored at -70°C .

11) PCR/Purification

- PCR was performed using Phusion High-Fidelity DNA Polymerase (Finnzymes Inc.) in 25 uL reactions with gene-specific, modified Solexa A1 and A2 primer pairs using standard reaction conditions and touchdown PCR in which the annealing temperature was decreased by 0.5 C every cycle, from 68 C to 60 C, at which 25 cycles were carried out. 20ul from each PCR reaction was run on a 2% super fine agarose gel (Amresco) for amplification confirmation and gel purification. PCR products were gel purified using Qiaex II Gel Extraction Kit (Qiagen) and eluted in 20 uL Qiagen EB. Pooled samples for sequencing were generated by combining 5ul of each of the 300 assays (5 genes x 60 samples/gene), ethanol precipitated and resuspended to 10nM in water (1% tris-buffered). The concentration of the pooled samples was determined using a NanoDrop. 20ul of 100uM custom sequencing primer and 20ul of 10nM of the pooled sample were sent to the Cornell Life Sciences Core Laboratories Center for Illumina/Solexa DNA sequencing.

Figure 1p
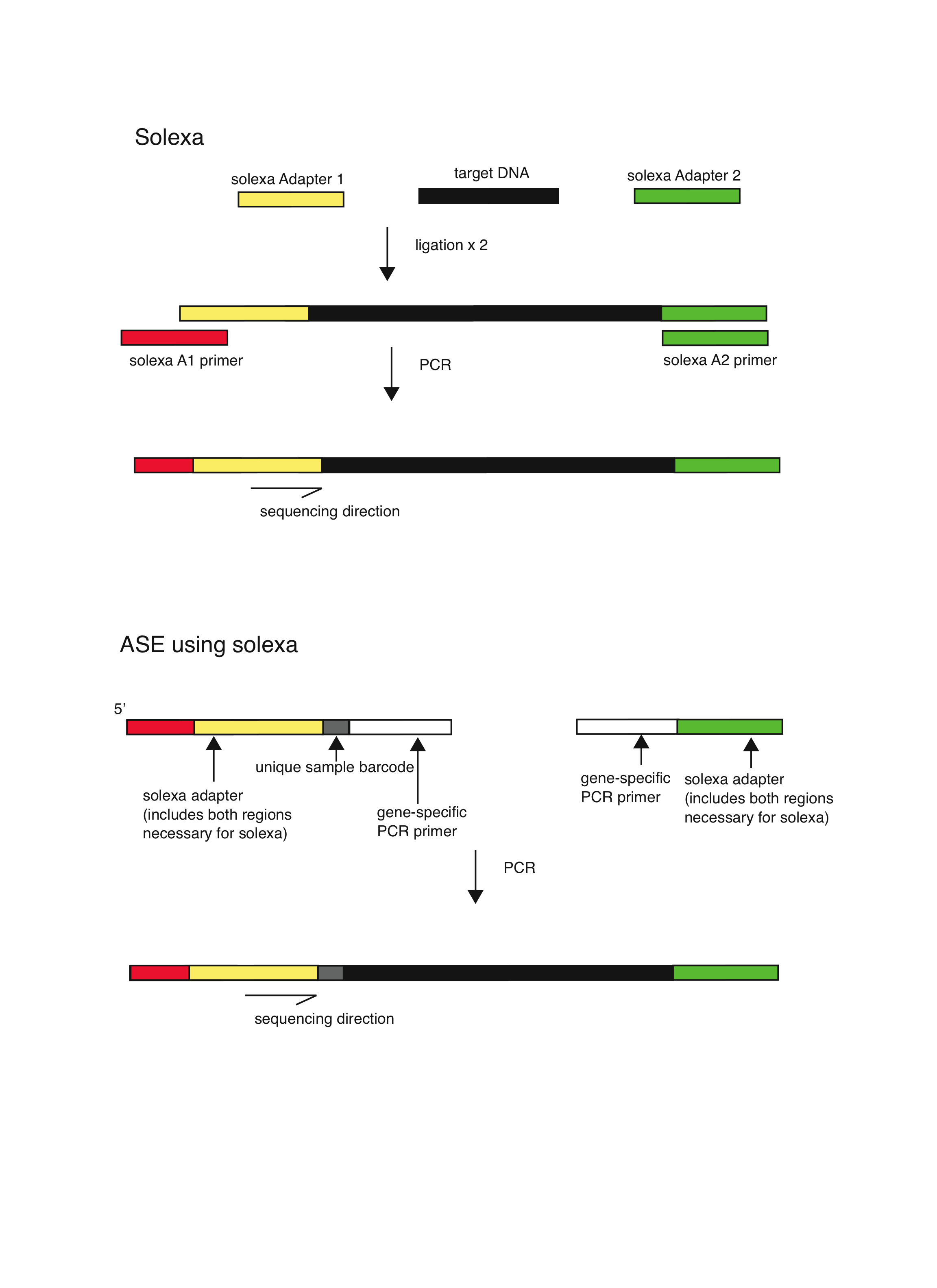

Supplement: Additional file 2 — Detailed protocol. "ase using solexa protocol " Detailed Protocol [file 1471-2164-10-422-S2.doc]
